# Alternative psychopharmacologic treatments for pediatric catatonia: a retrospective analysis

**DOI:** 10.3389/frcha.2023.1208926

**Published:** 2023-06-20

**Authors:** Joshua R. Smith, Isaac Baldwin, Tasia York, Carina Anderson, Trey McGonigle, Simon Vandekar, Lee Wachtel, James Luccarelli

**Affiliations:** 1 Division of Child and Adolescent Psychiatry, Department of Psychiatry and Behavioral Sciences, Vanderbilt University Medical Center at Village of Vanderbilt, Nashville, TN, United States; 2 Vanderbilt Kennedy Center, Vanderbilt University, Nashville, TN, United States; 3 Division of General Psychiatry, Department of Psychiatry and Behavioral Sciences, Vanderbilt University Medical Center, Nashville, TN, United States; 4 Department of Biostatistics, Vanderbilt University, Nashville, TN; 5 Kennedy Krieger Institute, Johns Hopkins School of Medicine, Baltimore, MD, United States; 6 Division of Child and Adolescent Psychiatry, Department of Psychiatry, Massachusetts General Hospital, Boston, MA, United States; 7 Department of Psychiatry, Harvard Medical School, Boston, MA, United States

**Keywords:** pediatric catatonia, electroconvulsive therapy, benzodiazepines, pediatric psychopharmacology, consult liaison

## Abstract

**Introduction:**

Pediatric catatonia is a highly co-morbid condition with treatment options often limited to electroconvulsive therapy (ECT) or lorazepam. However, lorazepam may not be readily available, and access to ECT is limited by restrictive legislation and stigma. This study aims to provide alternative treatment options for pediatric catatonia.

**Methods:**

The study involved a single-site retrospective analysis of a private university hospital in the southern United States. The study included patients under eighteen with catatonia who received psychopharmacologic treatment with an agent other than lorazepam. The patients were evaluated with the Bush-Francis Catatonia Rating Scale (BFCRS), Kanner Catatonia Severity Scale (KCS), and Kanner Catatonia Examination (KCE) at the time of initial evaluation and upon stabilization. A retrospective clinical global impressions-improvement (CGI-I) score was assigned by four authors.

**Results:**

102 pediatric patients diagnosed with catatonia were identified, and 31 met criteria for the study. 20 (65%) were white, 6 (19%) were Black, 4 (13%) were Hispanic, and 1 (3%) were Indian. Most patients (*N* = 18; 58%) were insured by Medicaid. The mean age at the time of catatonia diagnosis was 13.5 years. All patients were stabilized on either clonazepam or diazepam, with 21 (68%) requiring treatment with an additional medication of either an anti-epileptic, N-methyl-D-aspartate (NMDA) receptor antagonist, and aripiprazole or clozapine. Statistically significant reductions in the BFCRS [*t* = 11.2, df = 30, std = 6.3, *p* < 0.001, 95% CI = (7.8, 15.1)], KCS [*t* = 4.6, df = 38, *p* < 0.001, 95% CI = (12.0, 31.0)], and KCE [*t* = 7.8, df = 30, std = 1.8, *p* < 0.001, 95% CI = (1.9, 3.2)] were observed. For CGI-I the results showed that the estimated probability of observing a score better than no change (>4) is 0.976 [t.s. = 43.2, *p* < 0.001, 95% CI = (0.931,0.992)], indicating the average subject is expected to experience some improvement.

**Discussion:**

In conclusion, all patients responded to these treatments with improvement in their catatonic symptoms. Alternative pharmacologic interventions for catatonia, including benzodiazepines other than lorazepam, valproic acid, NMDA receptor antagonists, and atypical antipsychotics were safe and effective in treating catatonia in this population.

## 1. Introduction

Catatonia is a psychomotor disorder with affective domains and distinct physical examination findings. In adults, catatonia has been described in individuals with a range of psychiatric and medical conditions (1). Recently, evidence has suggested that catatonia may present differently in children, particularly those with developmental disorders (2, 3). Compared to catatonia in neurotypical adults, catatonia in children and those with co-morbid neurodevelopmental disorders (NDD) can present distinct symptoms and physical examination findings, which may increase the risk of a missed diagnosis. Examples of symptoms unique to pediatric catatonia include urinary incontinence, loss of previously acquired skills/communicative abilities, acrocyanosis, automatic compulsive movements, and schizophasia (4–6). Externalizing symptoms such as physical aggression, recurrent self-injury, and negativism may also be more common in pediatric and neurodevelopmental catatonia (7, 8), leading to difficulties obtaining a complete physical examination. These diagnostic challenges are of clinical concern as a missed diagnosis of catatonia may result in worsening/ongoing aggression and/or progression to malignant catatonia, a condition associated with autonomic instability and mortality rates as high as 10%–20% if left untreated (5). In addition, pediatric catatonia has been associated with a greater than sixty-fold higher risk of death than the general population (9). Thus, rapid identification and treatment of catatonia in children of diverse neurodevelopmental backgrounds is critical.

Per the 2023 consensus guidelines from the British Association for Psychopharmacology, high-dose lorazepam and electroconvulsive therapy (ECT) are considered the gold standard of catatonia treatment for adults and children (10). Despite ECT’s clinical effectiveness in catatonia, access is often limited due to a lack of provider availability, stigma, and state-dependent legislation restricting ECT access for children (11). During the international lorazepam shortage of 2022, providers faced significant limitations in therapeutic options for treating catatonia, particularly providers without ready access to ECT. Due to limited research of alternative treatments for pediatric catatonia, this critical shortage posed a significant challenge in managing this severe and life-threatening condition. Research in adults supports alternative psychopharmacologic treatments for catatonia, including N-methyl-D-aspartate (NMDA) receptor antagonists, anti-epileptics, aripiprazole, and clozapine (12). In addition, preliminary work supports the use of alternative benzodiazepines in pediatric catatonia (13, 14). However, in the pediatric literature, only three case reports have been published supporting alternative pharmacologic approaches other than ECT and benzodiazepines. These specific reports include one case of memantine use (15), and two supporting treatment with amantadine (16, 17). Overall, while many case reports are published that discuss the treatment of catatonia, there are few well-powered research studies (18), an issue that is doubly compounded in pediatric catatonia (5, 19). Thus, greater research in this area is urgently needed.

This study presents a single-site retrospective analysis of pediatric catatonia patients who were treated with medications other than lorazepam and assesses the safety and efficacy of such agents in the treatment of pediatric catatonia. Overall, we aim to expound on the current literature in the field of pediatric catatonia and provide alternative treatment options for providers managing this highly morbid condition.

## 2. Materials and methods

Strengthening the Reporting of Observational Studies in Epidemiology guidelines were followed in our study (20). Using the SlicerDicer software found within the Epic Systems electronic medical record (21), we conducted a single-site retrospective analysis of a private university hospital in the southern United States. Treatment settings included in this study were a pediatric medical inpatient unit, a pediatric inpatient psychiatric unit, or a psychiatric outpatient clinic specializing in treating catatonia in neurodevelopmental disorders. The study period was from 08/17/2021 to 11/17/2022. The inclusion criteria for this study were as follows: (1) receiving a diagnosis of catatonia per the fifth edition of the Diagnostic and Statistical Manual of Mental Disorders from a child and adolescent psychiatrist (22), (2) documented use of either the Bush-Francis Catatonia Rating Scale (BFCRS) (23) or Kanner Catatonia Rating Scale (KCRS) to confirm the diagnosis of catatonia at the time of initial evaluation and to assess for improvement at follow-up (24), (3) were under 18 years old at the time of diagnosis, and (4) received psychopharmacology treatment for catatonia with an agent other than lorazepam. Patients were excluded if their catatonia was managed with lorazepam monotherapy. To investigate how catatonia impacts individuals of varying backgrounds, data on age, Medicaid status, biological sex, race, ethnicity, psychiatric diagnoses, and psychopharmacologic history were collected. The institutional review board of Vanderbilt University Medical Center approved and oversaw this retrospective study (#220898) with a waiver of informed consent.

### 2.1. Symptom assessment

Catatonia was assessed using the BFCRS and/or KCRS for all patients. The KCRS was used in this study as it is designed to capture catatonic symptoms in individuals with varying neurodevelopmental backgrounds. Compared to the BFCRS, the KCRS places greater emphasis on reduced oral intake and externalizing behaviors, both symptoms of catatonia more common in pediatrics and those with NDDs. The KCRS includes a severity scale (KCS) and a standardized physical examination (KCE) (24). For patients seen via telemedicine, catatonia rating scales were calculated after observing parents and/or primary caregivers conduct the BFCRS, and KCE, and report specific symptoms over video (25).

### 2.2. Statistical analysis

For each patient included in the study, we obtained a retrospective clinical global impressions-improvement (CGI-I) score (26) assigned by the following authors: JRS, IB, TY, and CA. The CGI-I score was determined after each author reviewed the following components of the patient’s medical record: inpatient and outpatient progress notes, ECT procedural documentation, inpatient and outpatient admission/intake notes, and consult the documentation. Each author was blinded to the results determined by their co-authors; thus 4 separate retrospective CGI-I scores were computed for each patient. Interrater reliability of the retrospective CGI-I score was calculated using Gwet’s AC_2_. Using CGI-I data, we fit an ordinal GEE model to investigate the probability of observing improvement (a CGI-I score greater than (4) while accounting for intra-rater correlation. Baseline BFCRS, KCS, and KCE scores were compared to scores at the end of treatment using paired *t*-tests. All tests were 2-sided, with a prespecified significance threshold of *p* < 0.05, without correction for multiple testing. Clinical response was defined as an average retrospective CGI < 4 measured by all four raters.

## 3. Results

### 3.1. Case selection

A total of one hundred and two pediatric patients with a catatonia diagnosis were identified. Thirty-one patients (31%) met the criteria for our study. Our analysis identified 7 (23%) patients most recently evaluated by telemedicine. For 2 (6.5%) patients, the KCRS was not done during the initial evaluation. Thus, KCRS scores were retrospectively obtained based on data reported in the BFCRS and the subjective portions of the initial evaluation.

### 3.2. Patient demographics, diagnoses, psychopharmacologic medication history, and baseline speaking Status

A total of thirty-one pediatric patients with catatonia met the inclusion criteria. Sample demographics, insurance status, historic diagnoses, psychopharmacologic history, history of seizures, and baseline verbal ability can be found in Table 1. The mean age at the time of catatonia diagnosis was 13.5 years, with a median of 15 years and a standard deviation (SD) of 3.6. 20 (65%) patients were *white*, 6 (19%) were *Black*, 4 (13%) were *Hispanic*, and 1 (3.2%) were *Indian*. The majority of patients (*N* = 18; 58%) were insured by Medicaid. Diagnostically, 26 patients (84%) had a neurodevelopmental disorder diagnosis prior to the onset of catatonia. These include 20 (65%) with a diagnosis of autism spectrum disorder, and 16 (52%) met the criteria for autism with profound impairment based on symptomology per the Lancet Commission definitions of profound autism (27). 2 of 16 (13%) patients with profound autism were under the age cut-off of eight years but were included in this group due to the severe nature of their symptoms. At baseline, 15 patients (48%) experienced baseline impairments in their verbal ability, including 8 (26%) who were non-speaking and 7 (23%) who were minimally verbal based on recent definitions described in meta-analytic work by Koegel and colleagues (28). Four patients had known genetic diagnoses. Previous trials of psychiatric medications were common in the sample. As outlined in Table 1, 23 (80%) of patients had been previously prescribed psychiatric medication, with a mean of 4.5 and a median of 3 prior medication trials.

### 3.3. Clinical setting and acute factors leading to catatonia onset

Table 2 includes data regarding the clinical location where catatonia was first identified, along with acute factors leading to the onset of catatonia. 27 patients (87%) were diagnosed with catatonia in the inpatient setting, while 4 (13%) were diagnosed as outpatients. Of the 27 diagnosed as an inpatient, 26 of the 27 inpatients (96%) were first identified by the pediatric medical hospital’s child and adolescent psychiatry consult liaison (CAP-CL) service, and 1 of the 27 (3.7%) were identified in the inpatient psychiatric unit. 4 patients (13%) had an acute medical condition attributed to the onset of catatonia. These medical conditions included seronegative auto-immune encephalitis (*N* = 2; 6.5%), anti-NMDA receptor encephalitis (*N* = 1; 3.2%), and delirium (*N* = 1; 3.2%). In addition, 2 (6%) were prescribed a methylphenidate product to treat attention deficit hyperactivity disorder (ADHD) before admission. Notably, both patients were stimulant naïve at the time of methylphenidate administration. One patient developed catatonic symptoms after using cannabis. Psychiatric diagnoses associated with catatonia were present for 6 patients (19%). These include psychotic disorders (*N* = 4; 13%), bipolar 1 disorder (*N* = 2; 6.5%) 25 patients (81%) had a partial response to lorazepam (Supplementary Table S1, available online). With lorazepam treatment, 13 patients (42%) experienced breakthrough catatonic symptoms prior to the next lorazepam dose when dosed every six or every four hours.

Regarding additional acute precipitating factors, 9 (29%) patients experienced events described as stressful or traumatic by either the patient or their caregivers before the onset of catatonia. Specific examples include the following: 2 experienced the birth of a younger sibling, 2 were physically assaulted, 1 moved across the country with family, 1 developed catatonia following surgery to repair scoliosis, 1 experienced cardiac arrest resulting in delirium followed by catatonia, and 1 was placed in physical restraints while at school. Of these nine patients, 8 of 9 (88%) carried a historical diagnosis of intellectual disability. The only patient in this sample without a history of intellectual disability developed catatonia in the setting of cardiac arrest and delirium.

### 3.4. Acute psychopharmacologic treatments of pediatric catatonia

**FIGURE 1:**
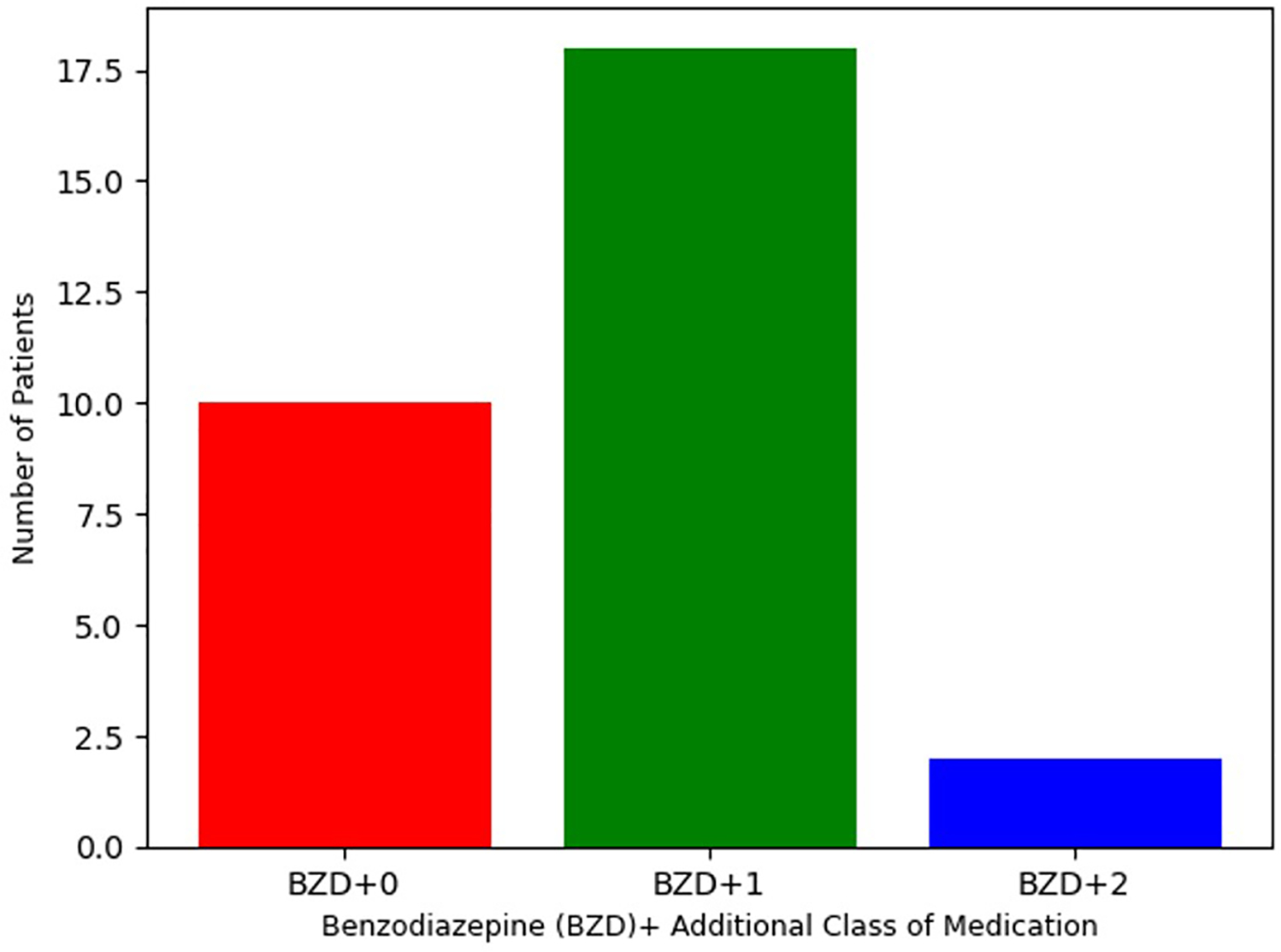
Number of medication classes in pediatric catatonia treatment.

As defined by inclusion criteria, all 31 patients were treated with either clonazepam or diazepam. As reported in Table 3, clonazepam was the most commonly prescribed benzodiazepine, with 27 patients (87%) receiving this agent (mean = 7.0 mg, SD = 7.1). The remaining 4 patients (13%) received diazepam (mean = 161 mg, SD = 28 mg). Despite the high dosage of benzodiazepines, no adverse effects were reported, including respiratory suppression. Per Figure 1, 10 patients (32%) were treated only with clonazepam or diazepam. However, incomplete treatment response was experienced by 21 (68%) patients in this sample. Therefore, alternative pharmacologic treatments were also initiated based on therapeutic algorithms supported by the adult catatonia literature including (Table 3) NMDA receptor antagonists (*N* = 13, 42%), anti-epileptics (*N* = 6, 19%), and aripiprazole or clozapine (*N* = 4, 13%) (12). 19 (61%) received were prescribed one additional class of medication, and 2 (6.5%) received medications from three separate classes.

Significant aggression and hyperactivity consistent with treatment-refractory excited catatonia was present for 3 patients (10%). These individuals required admission to the pediatric intensive care unit, where they were treated with infusions of midazolam and dexmedetomidine. The clinical course of these patients is described in Supplementary Table S2, available online. Following treatment with intravenous infusions of midazolam and dexmedetomidine, all three patients demonstrated significant clinical improvement and were transitioned to oral benzodiazepines. In total, 9 patients (29%) were also treated with ECT. Among ECT recipients, 8 were treated with two alternative pharmacologic agents, and 1 was managed with one. A summary of ECT treatment can be found in Supplementary Table S3, available online.

### 3.5. Clinical outcome measures

**FIGURE 2:**
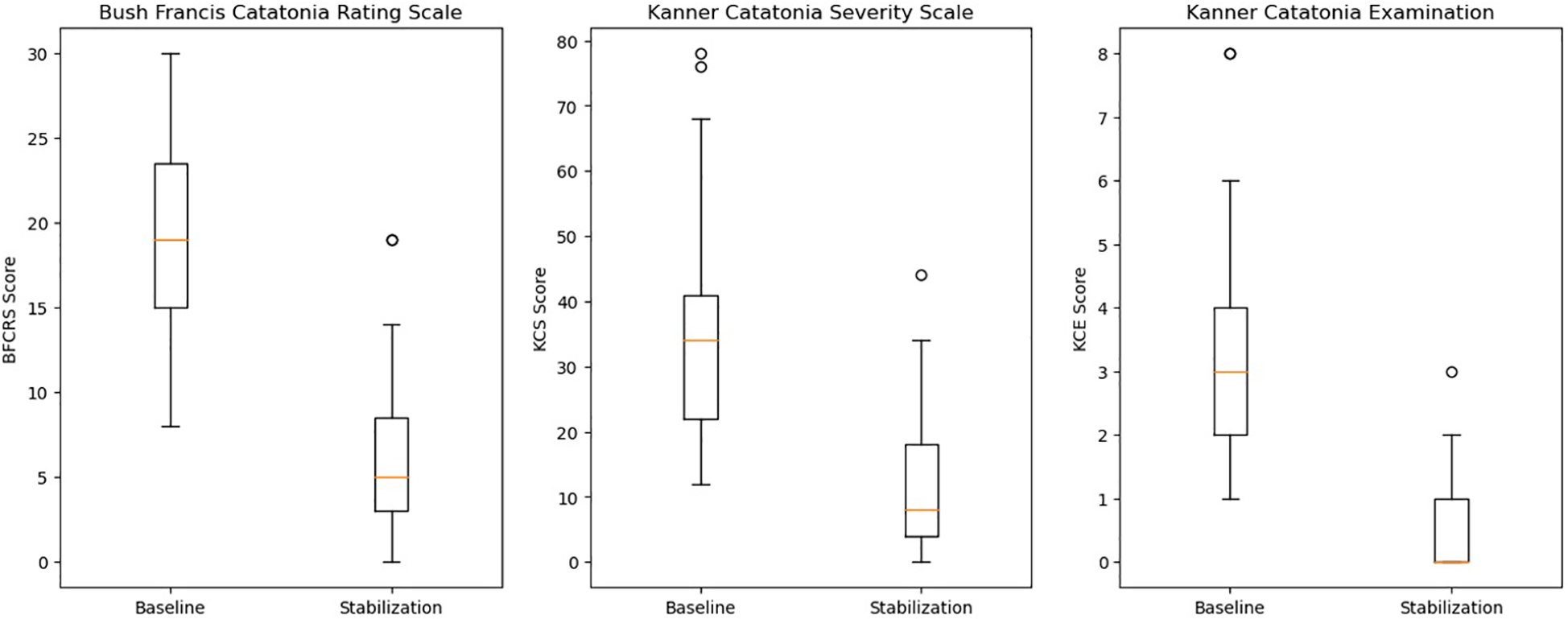
Comparison of catatonia outcome measures.

As seen in Figure 2, statistically significant reductions in the BFCRS [*t* = 11.2, df = 30, std = 6.3, *p* < 0.001, 95% CI = (7.8, 15.1)], KCS [*t* = 4.6, df = 38, *p* < 0.001, 95% CI = (12.0, 31.0)], and KCE [*t* = 7.8, df = 30, std = 1.8, *p* < 0.001, 95% CI = (1.9, 3.2)] were observed. For CGI-I scores, we tested the ordinal GEE model intercepts, which represent the log odds that a subject falls into a scoring category greater than a given score. However, for the sake of interpretability, we transformed these log odds into the corresponding probabilities of occurrence. We found that both intercepts indicating a score greater than 4 or 3 were significant. The results showed that the estimated probability of observing a score better than no change (>4) is 0.976 [t.s. = 43.2, *p* < 0.001, 95% CI = (0.931,0.992)], indicating the average subject was expected to experience some improvement. Additionally, it was also very likely for a subject to experience “much improvement” with the estimated probability of receiving a CGI-I score greater than 3 of 0.863 [t.s. = 33.6, *p* < 0.001, 95% CI = (0.772,0.921)]. Moreover, we found high inter-rater reliability with a Gwet’s AC_2_ of 0.809 [95% CI = (0.726,0.893)]. Lastly, only 4 (13%) of patients were readmitted for psychiatric symptomology throughout the study period.

## 4. Discussion

This manuscript is a continuation of a brief report which discussed five pediatric patients with profound autism and hyperactive catatonia who received alternative psychopharmacologic interventions. In this brief report, we found that the use of benzodiazepines other than lorazepam, valproic acid, and NMDA receptor antagonists was safe and effective in treating catatonia in this population (29). Expanding on this previous work, in a retrospective cohort of 31 patients receiving alternative pharmacologic treatments for catatonia other than lorazepam, response (defined as an average retrospective CGI < 4 measured by all four raters) was observed in all 31 patients. Prior to initiating these treatments, 25 (81%) demonstrated a partial treatment response using lorazepam.

In our sample, all patients who experienced lorazepam partial response or breakthrough symptoms were stabilized on benzodiazepines. Among benzodiazepines, 27 patients (87%) had catatonia stabilized with clonazepam and 4 (13%) with diazepam. Benzodiazepines are positive allosteric modulators of the GABA-A receptor, but individual agents differ in pharmacokinetic properties, including half-life, receptor binding affinity, and lipid solubility (30). As a result, different benzodiazepines may be expected to have differing efficacies in the treatment of catatonia. This is supported by a crossover trial in adults comparing lorazepam and oxazepam, where the overall response was similar between the two agents, but with differing profiles of symptom relief and superiority of lorazepam on the second day of administration (31). Treatment with multiple agents was common as 21 (68%) of patients required treatment with a medication of a differing class for stabilization. These included NMDA receptor antagonists (*N* = 13, 42%), anti-epileptics (*N* = 6, 19%), and aripiprazole or clozapine (*N* = 4, 13%). 3 (10%) patients required midazolam and dexmedetomidine infusions for stabilization before transitioning to oral benzodiazepine. Our group reviewed two of these three PICU cases in our preliminary report addressing alternative psychopharmacologic interventions in pediatric catatonia (29).

One possible explanation for the high degree of lorazepam partial response (*N* = 25, 81%) in this study population is cortical hyperplasticity, associated with developmentally appropriate cortical growth in children (32), and is a leading neurobiological theory regarding the neurobiology of autism. Specifically, it is hypothesized that an excitatory:inhibitory (E:I) imbalance is present in autism, resulting in cortical hyperactivity is present in autism which may be indicative of GABAergic dysfunction and/or hyperplasticity due to impairment of long-term cortical plasticity mechanisms mediated by the NMDA receptor (33–35). Recent diagnostic work in transcranial magnetic stimulation of intellectually capable persons with autism (AIC) has reported enhanced cortical modulation, indicative of cortical hyperplasticity (35, 36). Furthermore, recent preliminary magnetoencephalographic research has reported greater E:I imbalance in biologically male patients with autism and co-morbid intellectual disability (AID) compared to AIC (37), suggests a direct correlation between the degree of E:I imbalance and cognitive impairment. These findings, along with the potential role of GABAergic signaling dysfunction in catatonia (5) and baseline cortical hyperplasticity observed in children (32), may explain why longer-acting benzodiazepines at high dosages were required to stabilize catatonia. Specifically, a hyperplastic cortex may rapidly acclimate to a relatively short-acting benzodiazepine such as lorazepam leading to inadequately managed catatonic symptomology (4, 35). Indeed, previous case reports have identified lorazepam tolerance as a possible complication in the treatment of catatonia for individuals with co-morbid intellectual disabilities (38, 39).

Clinically, a statistically significant benefit was observed in the following domains: BFCRS, KCS, KCE, and retrospective CGI-I. Moreover, only 4 (13%) of patients were psychiatrically readmitted over the study period. In comparison, psychiatric readmission for children ranges from 10%–30% for any diagnosis and nearly 32% for children with a psychotic disorder (40).

Diagnostically, 84% of patients in our cohort had a neurodevelopmental disorder, with 65% having a previous diagnosis of autism spectrum disorder. These findings may occur due to the study site’s inclusion of a neurodevelopmental psychiatry clinic specializing in catatonia and that the study population was identified in a large children’s hospital. However, the data does provide additional evidence pointing to a connection between catatonia, neurodevelopmental disorders, and co-morbid genetic syndromes documented in other reports (4, 41). 19% were diagnosed with psychiatric conditions which contributed to the onset of catatonia. These findings are especially relevant as identification From a socio-economic perspective, 58% of patients were enrolled in Medicaid, highlighting that the majority of these patients and families likely experience financial as well as medical and psychiatric challenges.

Historical research has attempted to determine what aspect of autism is most likely to increase the risk of catatonia in autism. Specifically, intellectual disability as a risk factor for catatonia development has been discussed in recent meta-analytic autism work (4) and other reports (42, 43). Alternatively, an expert opinion of catatonia in autism by Shah and colleagues has speculated that social-emotional relatedness may be a critical factor in catatonia development (44). Dhossche and Fink have speculated that trauma and psychosocial stressors precipitate catatonia in children (45, 46). This is consistent with our findings as 9 (29%) of the patients in our study reported highly stressful events prior to the onset of catatonia. While this retrospective sample does not allow for drawing specific conclusions regarding risk factors for catatonia in autism, future research should continue to explore the role of intellectual capacities and communicative abilities in the clinical presentation of catatonia. Given the degree of morbidity associated with catatonia for autistic individuals across the lifespan (2, 47), identification of specific risk factors is critical.

In addition, our study supports previous research connecting severe psychiatric and medical comorbidities to pediatric catatonia (3). 3 (10%) of patients had a medical diagnosis contributing to catatonia, including seronegative auto-immune receptor encephalitis, anti-NMDA receptor encephalitis, and delirium. Notably, while catatonia has been reported in pediatric auto-immune encephalitis (48) and cases of delirium in adult patients (49), to our knowledge, our study includes the first report of catatonia occurring after the onset of delirium in a pediatric patient. Moreover, we found that the majority of catatonic patients were identified in the inpatient setting by the CAP-CL team. Given the interface of psychiatric and medical care in catatonia, CAP-CL providers have long considered diagnosis and treatment of catatonia within the scope of their practice (7). However, this very high identification rate by CAP-CL providers observed in our study further emphasizes the importance of education regarding pediatric catatonia in CAP-CL. Moreover, due to the high morbidity and mortality associated with pediatric catatonia, this represents a possible area of high-impact clinical intervention for CAP-CL providers (3, 5, 9).

We also found one patient who developed catatonia after using cannabis and two others who developed catatonia after initiating methylphenidate in the treatment of ADHD. While catatonia has been reported as a potential complication of cannabis use in adolescents (50, 51), to the authors’ knowledge, this is the first report of catatonia days after initiating methylphenidate products to treat ADHD. The patients were biological males six and nine years of age, presenting to pediatric medicine and managed by the CAP-CL. The six-year-old patient carried diagnoses of autism and ADHD and received methylphenidate for three weeks before catatonia onset. The nine-year-old had a previous ADHD diagnosis and no other psychiatric comorbidities. This patient became catatonic within one day of methylphenidate initiation. These cases, the high rate of catatonia diagnoses in the pediatric medical hospital, and previous reports connecting cannabis use and catatonia highlight the need for CAP-CL psychiatrists to include robust systematic substance use screening in their regular clinical practice (7, 52).

In the clinical assessment of pediatric catatonia, the identification of neurodevelopmental disorders, autoimmune conditions, delirium, substance exposure, and psychiatric illness as possible causes of catatonia is critical. When present and identified, treating the underlying condition resulting in catatonia is a critical step to ensure recovery and remission of catatonia symptoms. However, specific causes of catatonia can be elusive and may not present until months or years after the acute onset of catatonia (53). In neurodevelopment disorders such as autism, the core symptoms of the disorder cannot be managed pharmacologically, limiting a clinician’s ability to address the underlying cause (4). Such complications highlight the importance of a comprehensive medical workup in acute catatonia as identification and treatment of an underlying condition may significantly impact a patient’s morbidity and long-term prognosis (10, 12).

Strengths of the study include a large sample size for a study of pediatric catatonia and broad inclusion criteria allowing for a description of treatment in children with a range of baseline diagnoses. As the study population is derived from a specialist pediatric hospital and NDD clinic, there are high rates of NDDs that may not be generalizable to the overall population of children with catatonia. Our study primarily focuses on the acute management of pediatric catatonia, without delving into the treatment of potentially causative underlying conditions. The authors recommend that future research explore this area by obtaining and analyzing longitudinal data once the acute phase of catatonia has resolved. Moreover, this study utilized the KCRS and BFCRS for catatonia assessment (24). Neither of these scales have been specifically validated in the pediatric population, and the BFCRS is not designed for individuals with NDDs. Additionally, seven patients were most recently evaluated by telemedicine, and the accuracy of remote assessment of catatonia has not been studied. The retrospective nature of CGI-I scoring is an additional limitation in our study. To mitigate this, we blinded authors who provided CGI-I scores and reported the degree of inter-rater reliability. Another potential limitation is the possibility of inaccuracies in medical records, an issue inherent to our study design. ECT was also used in 9 cases, which likely improved overall clinical outcomes for these patients. Therefore, our ability to fully determine the efficacy of pharmacologic interventions in these cases is limited. There is also the potential for bias in our study, as the BFCRS and KCRS were used as part of clinical care and thus, were unblinded. Lastly, due to the limited verbal ability of 15 (48%) patients in our study, the full scope of side effects was difficult to determine.

Overall, there is a high risk of morbidity and mortality associated with pediatric catatonia (3, 5, 9), which necessitates prompt screening and treatment. Our manuscript provides data supporting the safe and effective use of alternative psychopharmacologic agents in treating pediatric catatonia. Future research should address possible risk factors of pediatric catatonia in NDD populations, work to validate the KCRS and determine if additional catatonia symptoms are specific to a given population, and determine if other psychopharmacologic agents may be of therapeutic benefit, ideally with controlled trials.

## Supplementary Material

Table S1

Table S2

Table S3

## Figures and Tables

**TABLE 1:** Patient demographics, diagnoses, psychopharmacologic medication history, and speaking status.

Demographics					
Biological sex and age		Ethnicity		Insurance coverage	
Male	19/31 (59%)	*White*	20/31 (65%)	Medicaid	18/31 (58%)
Female	13/31 (41%)	*Black*	6/31 (19%)	Private Insurance	13/31 (42%)
Mean Age	Mean = 13.5 years, SD=3.6	*Hispanic*	4/31 (13%)		
		*Indian*	1/31 (3%)		
**Historic diagnoses and psychopharmacologic trials**					
**Neurodevelopmental diagnoses**		**Genetic diagnoses**		**Psychopharmacologic medication history**	
Total with neurodevelopment comorbidity	26/31 (84%)	*NBEA* variant	1/31 (3%)	Total number of patients who were previously prescribed psychiatric medications	24/31 (80%)
Autism with Profound Impairment	16/31 (52%)	Fragile × Syndrome	1/31 (3%)	Mean and median number of failed psychiatric medications	Mean = 4.5, Median = 3 (Min = 1, Max = 14, SD = 3.6)
Autism without Profound Impairment	4/31 (13%)	22q11.2 Deletion Syndrome	1/31 (3%)	Mean number of failed second-generation antipsychotics	Mean = 1.5 (Min = 0, Max = 5, SD = 1.3)
Intellectual Disability Disorder	4/31 (13%)	Trisomy 10	1/31 (3%)	Mean number of failed first-generation antipsychotics	Mean = 0.3 (Min = 0, Max = 2, SD = 0.6)
Unspecified Neurodevelopmental Disorder	2/31 (6%)			Mean number of failed mood-stabilizers	Mean = 0.4 (Min = 0, Max = 2, SD = 0.6)
				Mean number of failed NMDA antagonists	Mean = 0.17 (Min = 0, Max = 1, SD = 0.4)
**Seizure history and baseline verbal ability**					
**History of seizures**		**Baseline verbal ability**			
Total number of patients with a history of seizures	10/31 (32%)	Non-speaking	8/31 (26%)		
		Minimally verbal	7/31 (23%)		

**TABLE 2:** Clinical setting and acute factors leading to catatonia onset.

Clinical setting where catatonia was diagnosed	
Inpatient	27/31 (87%)
Outpatient	4/31 (13%)
**Inpatient setting where first diagnosed with catatonia**	
Inpatient pediatric medicine by CAP consult-liaison psychiatry	26/27 (96%)
Inpatient pediatric psychiatry	1/27 (4%)
**Acute medical condition at the time of catatonia diagnosis and treatment**	
Seronegative autoimmune encephalitis	2/31 (6%)
Anti-NMDA receptor encephalitis	1/31 (3%)
Delirium	1/31 (3%)
**Substance exposure preceding onset of catatonia**	
Methylphenidate	2/31 (6%)
Cannabis	1/31 (3%)
**Psychiatric co-morbidities contributing to onset of catatonia**	
Total with previous psychiatric diagnoses	6/31 (19%)
Unspecified schizophrenia spectrum and other psychotic disorder	3/31 (10%)
Bipolar disorder, type 1	2/31 (6%)
Childhood-onset schizophrenia	1/31 (3%)
**Stressful or traumatic event preceding onset of catatonia**	
Total number of patients with traumatic exposure prior to catatonia onset	9/31 (29%)

**TABLE 3:** Summary of alternative acute psychopharmacologic treatment of pediatric catatonia

Pharmacologic Intervention	Medication class	Total number of patients using the medication	Mean (SD) total daily dosage	Median total daily dosage	Range of total daily dosage
Clonazepam	Benzodiazepine	27/31 (87%)	7 mg (7.1)	6.9 mg	0.8 mg-24 mg
Diazepam	Benzodiazepine	4/31 (13%)	161.3 mg (28.4)	172.5 mg	120 mg-180 mg
Memantine	NMDA Receptor Antagonist	12/31 (39%)	17.7 mg (6.2)	5.8 mg	5 mg-25 mg
Amantadine	NDMA Receptor Antagonist	1/31 (3%)	150 mg	-	-
Valproic Acid	Anti-epileptic	4/31 (13%)	1050 mg (665.8)	576.6 mg	500 mg-2000 mg
Oxcarbazepine	Anti-epileptic	2/31 (6%)	300 mg	-	-
Aripiprazole	Atypical Antipsychotic	3/31 (10%)	10 mg (5)	5 mg	5 mg-15 mg
Clozapine	Atypical Antipsychotic	1/31 (3%)	300 mg	-	-

## Data Availability

The raw data supporting the conclusions of this article will be made available by the authors, without undue reservation.

## Abbreviations

NDD: neurodevelopmental disorder
ECT: electroconvulsive therapy
NMDA: N-methyl-D-aspartate
BFCRS: bush-francis catatonia rating scale
KCRS: kanner catatonia rating scale
KCS: kanner catatonia severity
KCE: kanner catatonia examination
CGI-I: clinical global impressions-improvement
SD: standard deviation
CAP-CL: child and adolescent psychiatry consult-liaison
ADHD: attention deficit hyperactivity disorder
EI: excitatory inhibitory
AIC: intellectually capable persons with autism
AID: autism with intellectual disability
