# Supplementary material for: Alternative psychopharmacologic treatments for pediatric catatonia: a retrospective analysis
Source: Front Child Adolesc Psychiatry. 2023 Jun 20;2:1208926. doi: 10.3389/frcha.2023.1208926 (PMC10312099; doi:10.3389/frcha.2023.1208926)
Supplement: Supplementary file 1 [file Presentation1.pptx]

## Slide 1
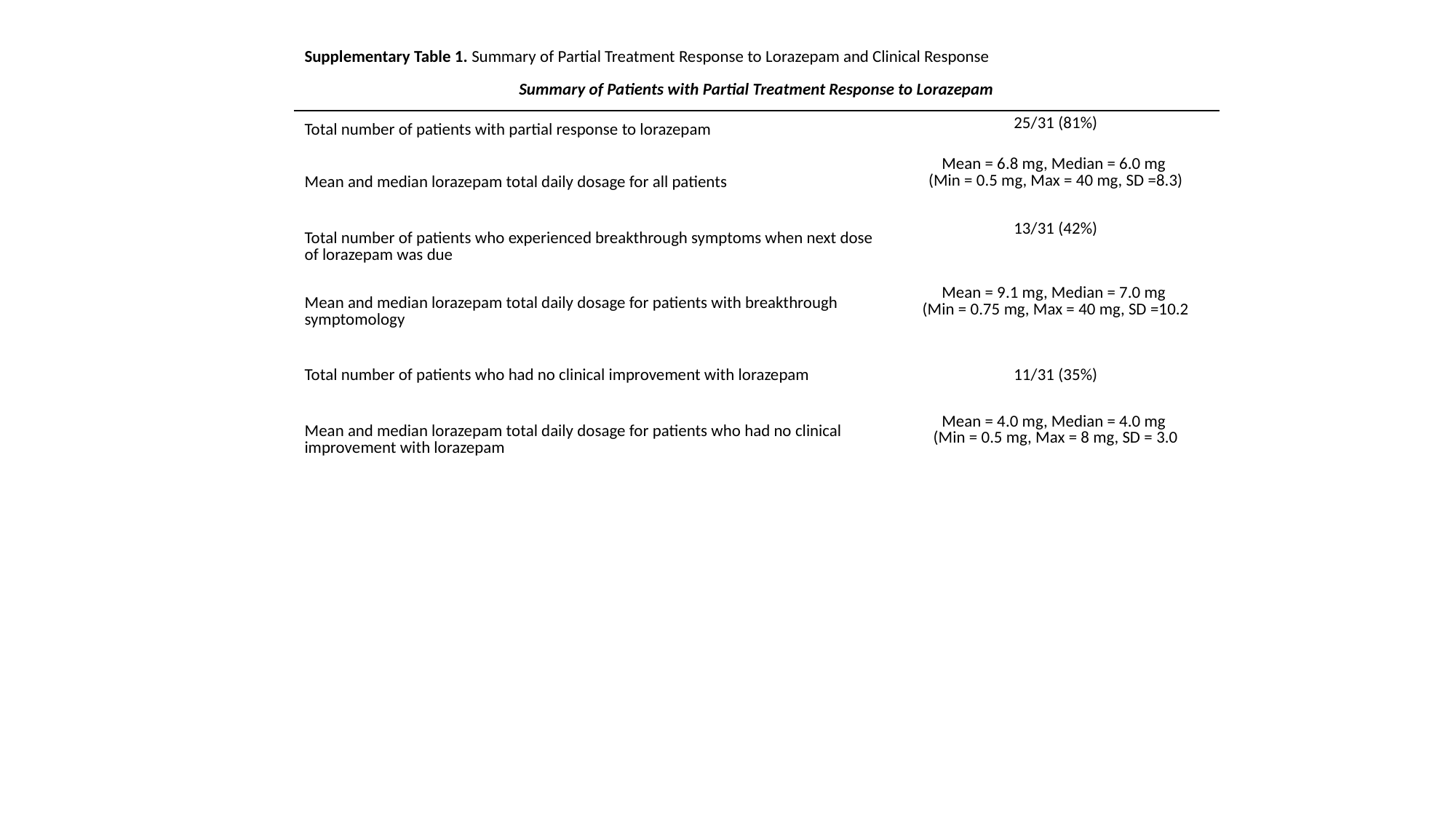

| Supplementary Table 1. Summary of Partial Treatment Response to Lorazepam and Clinical Response | |
| --- | --- |
| Summary of Patients with Partial Treatment Response to Lorazepam | |
| Total number of patients with partial response to lorazepam | 25/31 (81%) |
| Mean and median lorazepam total daily dosage for all patients | Mean = 6.8 mg, Median = 6.0 mg (Min = 0.5 mg, Max = 40 mg, SD =8.3) |
| Total number of patients who experienced breakthrough symptoms when next dose of lorazepam was due | 13/31 (42%) |
| Mean and median lorazepam total daily dosage for patients with breakthrough symptomology | Mean = 9.1 mg, Median = 7.0 mg (Min = 0.75 mg, Max = 40 mg, SD =10.2 |
| Total number of patients who had no clinical improvement with lorazepam | 11/31 (35%) |
| Mean and median lorazepam total daily dosage for patients who had no clinical improvement with lorazepam | Mean = 4.0 mg, Median = 4.0 mg (Min = 0.5 mg, Max = 8 mg, SD = 3.0 |
