# Supplementary material for: Alternative psychopharmacologic treatments for pediatric catatonia: a retrospective analysis
Source: Front Child Adolesc Psychiatry. 2023 Jun 20;2:1208926. doi: 10.3389/frcha.2023.1208926 (PMC10312099; doi:10.3389/frcha.2023.1208926)
Supplement: Supplementary file 2 [file Presentation2.pptx]

## Slide 1
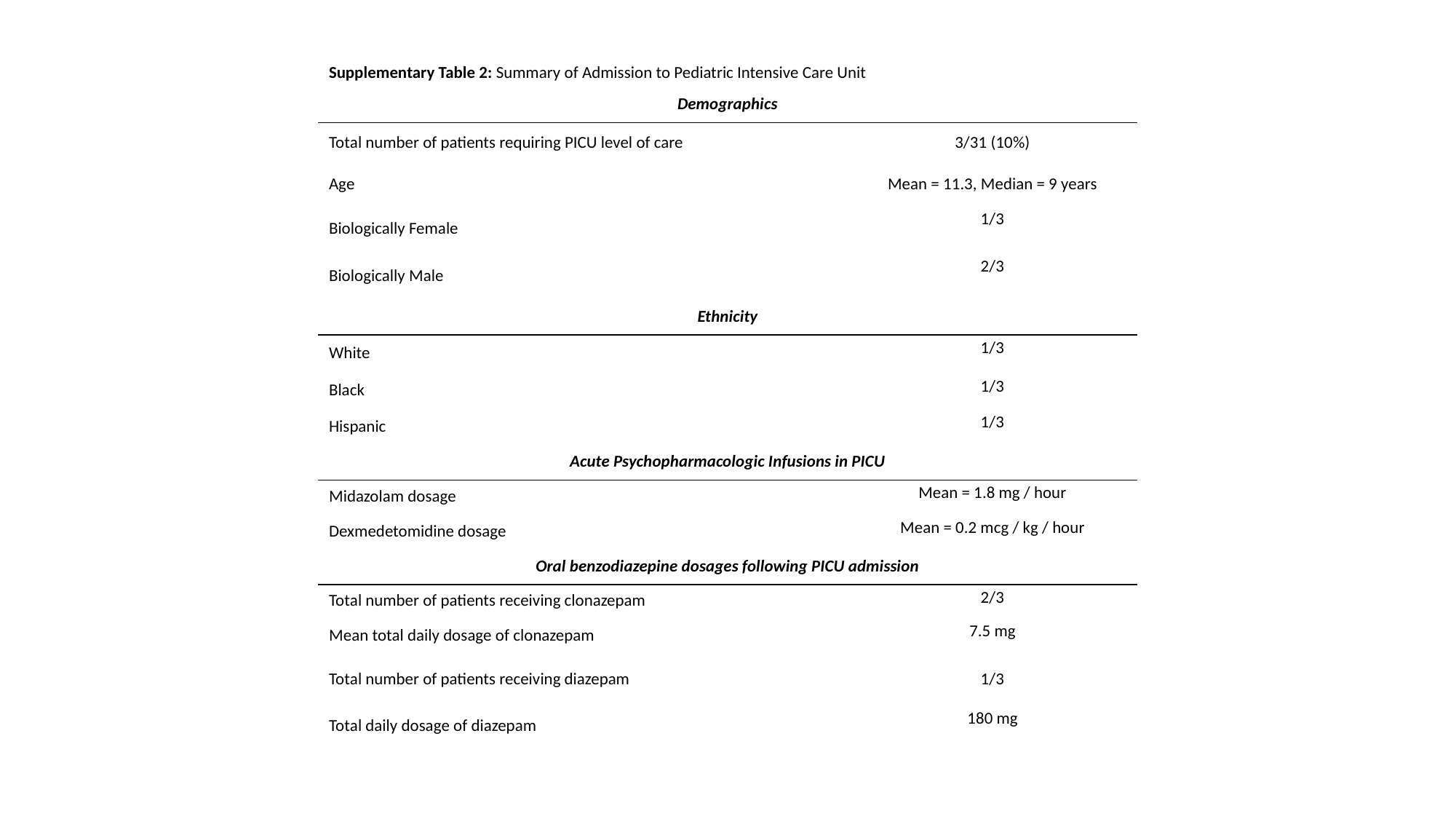

| Supplementary Table 2: Summary of Admission to Pediatric Intensive Care Unit | |
| --- | --- |
| Demographics | |
| Total number of patients requiring PICU level of care | 3/31 (10%) |
| Age | Mean = 11.3, Median = 9 years |
| Biologically Female | 1/3 |
| Biologically Male | 2/3 |
| Ethnicity | |
| White | 1/3 |
| Black | 1/3 |
| Hispanic | 1/3 |
| Acute Psychopharmacologic Infusions in PICU | |
| Midazolam dosage | Mean = 1.8 mg / hour |
| Dexmedetomidine dosage | Mean = 0.2 mcg / kg / hour |
| Oral benzodiazepine dosages following PICU admission | |
| Total number of patients receiving clonazepam | 2/3 |
| Mean total daily dosage of clonazepam | 7.5 mg |
| Total number of patients receiving diazepam | 1/3 |
| Total daily dosage of diazepam | 180 mg |
