# Supplementary material for: Alternative psychopharmacologic treatments for pediatric catatonia: a retrospective analysis
Source: Front Child Adolesc Psychiatry. 2023 Jun 20;2:1208926. doi: 10.3389/frcha.2023.1208926 (PMC10312099; doi:10.3389/frcha.2023.1208926)
Supplement: Supplementary file 3 [file Presentation3.pptx]

## Slide 1
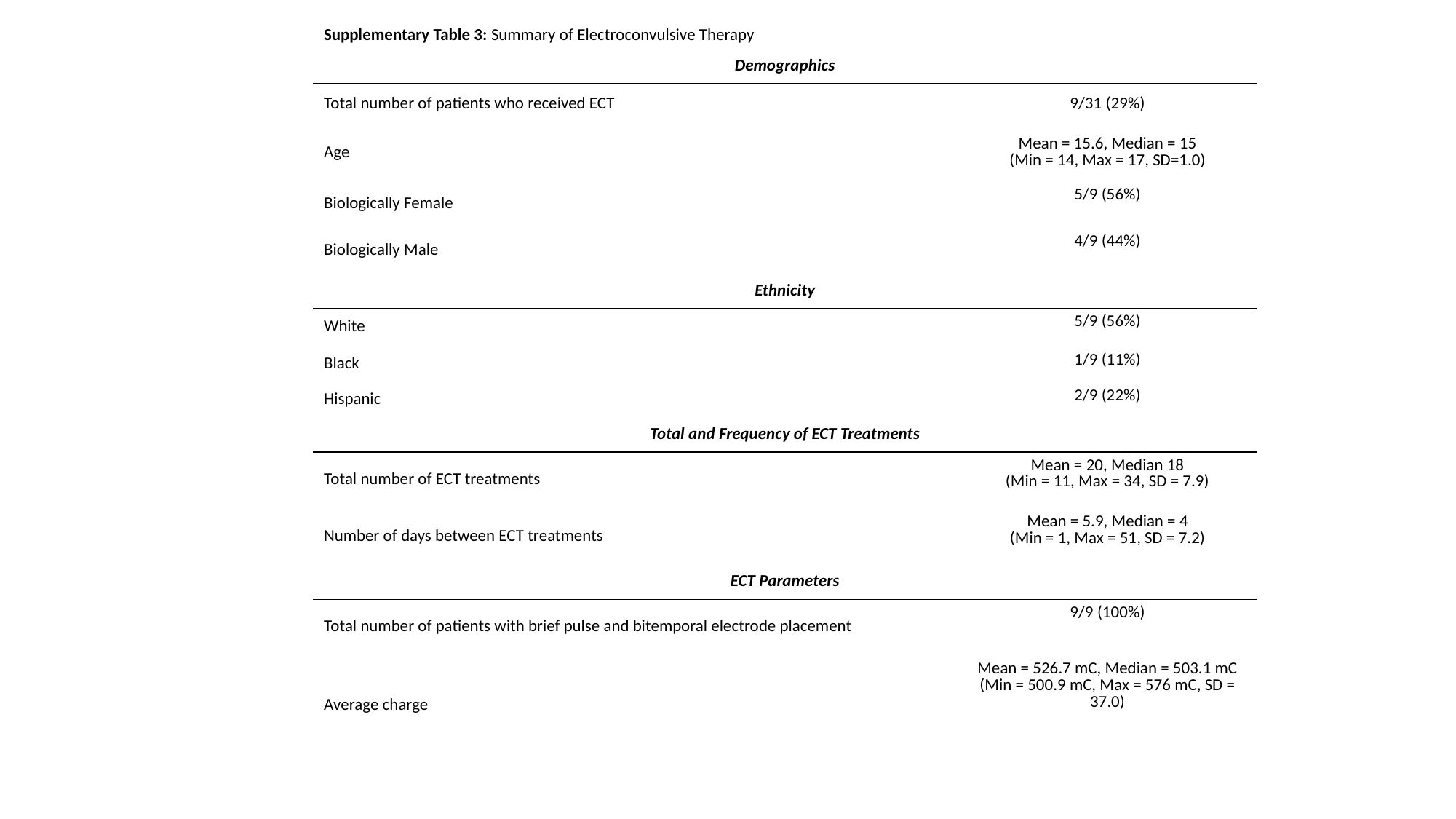

| Supplementary Table 3: Summary of Electroconvulsive Therapy | |
| --- | --- |
| Demographics | |
| Total number of patients who received ECT | 9/31 (29%) |
| Age | Mean = 15.6, Median = 15 (Min = 14, Max = 17, SD=1.0) |
| Biologically Female | 5/9 (56%) |
| Biologically Male | 4/9 (44%) |
| Ethnicity | |
| White | 5/9 (56%) |
| Black | 1/9 (11%) |
| Hispanic | 2/9 (22%) |
| Total and Frequency of ECT Treatments | |
| Total number of ECT treatments | Mean = 20, Median 18 (Min = 11, Max = 34, SD = 7.9) |
| Number of days between ECT treatments | Mean = 5.9, Median = 4 (Min = 1, Max = 51, SD = 7.2) |
| ECT Parameters | |
| Total number of patients with brief pulse and bitemporal electrode placement | 9/9 (100%) |
| Average charge | Mean = 526.7 mC, Median = 503.1 mC (Min = 500.9 mC, Max = 576 mC, SD = 37.0) |
